# Beautiful Chromos

**Published:** 1873-07

**Authors:** 


*Beautiful Chromos.*

Orange Judd & Co., publishers of that magnificent weekly, the *Hearth and Home*, and the *American Agriculturist*, have sent us two splendid chromos—the "Strawberry Girl" and "Mischief Brewing." These exquisite chromos are worth the price of subscription to these journals, but subscribers can get them free by sending for *Hearth and Home* (a magnificent illustrated weekly) and *American Agriculturist*—four dollars each. Subscription to either entitles to a chromo. Address Orange Judd & Co., 245 Broadway, New York.

